# Correction: Theoretical insights into site-specific heavy-atom effects on MR-TADF emitters: modulation of spin–orbit coupling and color purity

**DOI:** 10.1039/d6sc90086k

**Published:** 2026-04-17

**Authors:** Shi-Jie Ge, Jian-Rong Wu, Zuo-Quan Jiang

**Affiliations:** a State Key Laboratory of Bioinspired Interfacial Materials Science, Institute of Functional Nano & Soft Materials (FUNSOM), Soochow University Suzhou, 215123 Jiangsu PR China zqjiang@suda.edu.cn

## Abstract

Correction for ‘Theoretical insights into site-specific heavy-atom effects on MR-TADF emitters: modulation of spin–orbit coupling and color purity’ by Shi-Jie Ge *et al.*, *Chem. Sci.*, 2026, https://doi.org/10.1039/d6sc00582a.

The authors regret two mistakes in Fig. 1. Specifically, the cited *k*_RISC_ data range was incorrectly given as 10^−4^–10^−6^ s^−1^. The correct range is 10^4^–10^6^ s^−1^. The FWHM of BNSeSe in the solution state should be 38 nm instead of 48 nm.

The corrected version of Fig. 1, with the intended *k*_RISC_ values for BNSeSe, SSeQ and Se-SFBN and the correct FWHM of BNSeSe, is given here and replaces that included within the original publication.

**Fig. 1:**
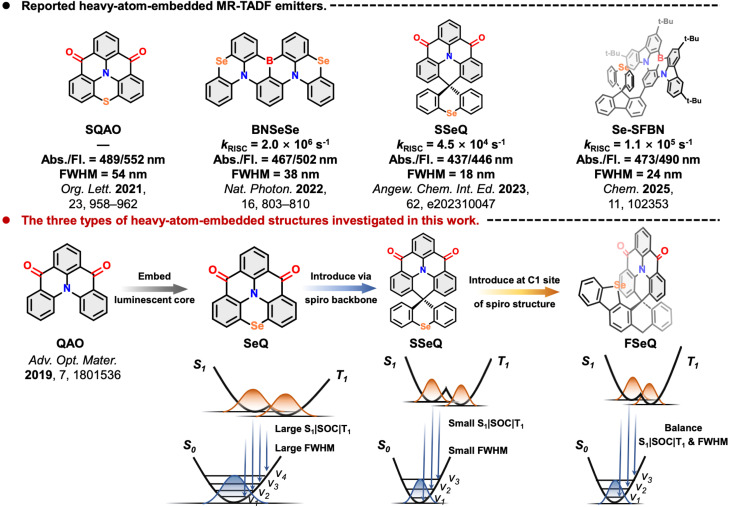
Reported heavy-atom-embedded MR-TADF emitters and the three types of heavy-atom-embedded structures investigated in this work.

The Royal Society of Chemistry apologises for these errors and any consequent inconvenience to authors and readers.

